# Empowering People with Disabilities in Smart Homes Using Predictive Informing †

**DOI:** 10.3390/s25010284

**Published:** 2025-01-06

**Authors:** Marko Periša, Petra Teskera, Ivan Cvitić, Ivan Grgurević

**Affiliations:** University of Zagreb, Faculty of Transport and Traffic Sciences, Vukelićeva 4, 10000 Zagreb, Croatia; petra.teskera@fpz.unizg.hr (P.T.); ivan.cvitic@fpz.unizg.hr (I.C.)

**Keywords:** accessibility, artificial intelligence, assistive technology, ambient assisted living, navigation

## Abstract

The possibilities of the Ambient Assisted Living (AAL)/Enhanced Living Environments (ELE) concept in the environment of a smart home were investigated to improve accessibility and improve the quality of life of a person with disabilities. This paper focuses on the concept of predictive information for a person with disabilities in a smart home environment concept where artificial intelligence (AI) and machine learning (ML) systems use data on the user’s preferences, habits, and possible incident situations. A conceptual mathematical model is proposed, the purpose of which is to provide predictive user information from defined data sets. This paper defines the taxonomy of communication technologies, devices, and sensors in the environment of the user’s smart home and shows the interaction of all elements in the environment of the smart home. Through the integration of assistive technologies, it is possible to adapt the home to users with diverse types of disabilities and needs. The smart home environment with diverse types of sensors whose data are part of sets defined by a mathematical model is also evaluated. The significance of establishing data sets as a foundation for future research, the development of ML models, and the utilization of AI is highlighted in this paper.

## 1. Introduction

According to the World Health Organization (WHO), an estimated 2.2 billion people have some form of vision impairment (distance or near), while 1.3 billion people have a significant vision disability, representing 16% of the world’s population. Vision loss can affect a person’s ability to perform daily activities and affects people of all ages. However, the majority of people with vision impairment and blindness are over 50 years old [1]. The implementation of assistive technologies can range from physical products such as wheelchairs, glasses, prosthetic limbs, white canes, and hearing aids to modern IT solutions and services such as speech recognition, smart home management, and the like. With the aging of the global population and the increase in non-communicable diseases, it is estimated that around 3.5 billion people will need assistive technology by 2050. Statistical data predicts that the global number of smart home users in the smart home market will continuously increase between 2023 and 2028 by a total of 424.5 million users (+117.69%), which is an indicator of a trend that can certainly affect the possibility of applying the smart home environment for people with visual impairments [2]. Everyday needs are an integral part of a person’s life that affect its quality. The home and its environment, and the contents found in it certainly have a significant impact on the quality of life. The development of information and communication technologies (ICT) and services opens the field of creating a smart home, which with its functionalities can raise the level of quality of life of the person living in it. Many years of work, as well as numerous pieces of research conducted in the domain of assistive technologies and their adaptation to people with visual impairments and other types of impairments, have shown that this field requires the development of services tailored to the requirements of users. The above is based on the fact that not all people have the same knowledge about the use of assistive technologies, therefore, the individual adaptation of services is the basis for better future application.

Using the methods of analysis and synthesis, information and data from previous research were collected and a taxonomy of communication technologies and services was defined. Also, using the above methods, data sets were defined that are the basis for the work of the mathematical model. The observation method was used to collect information (phenomena and processes) surrounding a blind and visually impaired person who is in a smart home environment.

The aim of this paper is to highlight the complex interdependence of the different elements of a smart home system and underscore the importance of defining data sets and creating user profiles depending on the type of impairment of the user.

## 2. Methodology

**Figure 1:**
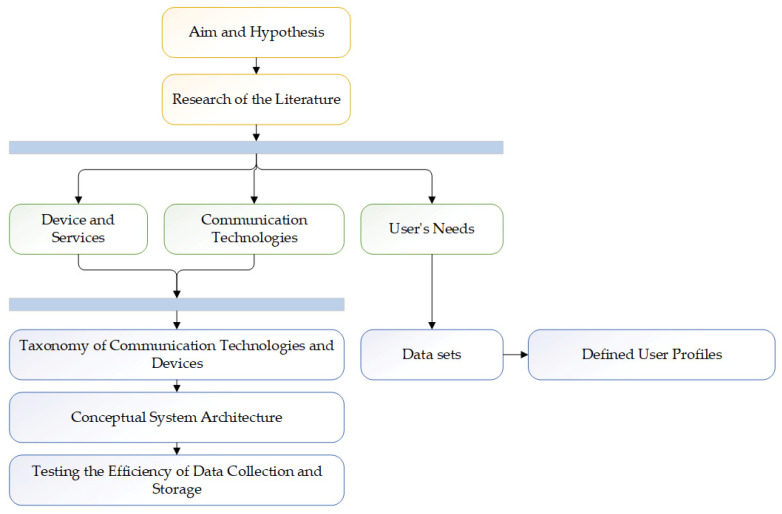
Research methodology.

Today’s technological development in the field of assistive technology and the environment of people with disabilities brings significant changes in the way people with disabilities live and participate in everyday life [3]. Figure 1 shows the research methodology in this paper. The research hypothesis is as follows: based on defined data sets, it is possible to create user profiles of user behaviour for the purpose of predictive information. For this purpose, the literature covering the development and application of assistive technologies (devices and services) was researched, the technical characteristics of communication technologies that can work in the IoT environment of a smart home were investigated, and user needs depending on the type of user impairment were also investigated.

Based on the methods used in the research, a conceptual architecture of the system will be presented, the aim of which is to show the interaction of data collection, processing, and storage. The defined data sets are presented mathematically and represent the basis for the development of machine learning (ML) models and the creation of a user knowledge base. Based on this, it is possible to develop user profiles necessary for predictive user information using artificial intelligence (AI). Predictive information works in such a way that data and information about user needs are collected daily in the smart home environment and personalized user information is created using AI.

### 2.1. Literature Review

Assistive technologies are not representation of only information and communication devices and systems, but also services aimed at improving the functional abilities of people with diverse types of disabilities [4]. Having a disability can impact a person’s ability to conduct daily tasks and fully participate in various environments, including their own home. However, everyday activities within their home such as cooking, cleaning, safe movement around the home, or health monitoring can be integrated into a smart home environment where users can control individual devices [5].

A smart home can be defined as the interaction of information and communication technologies and services with the user through the home network to create better living conditions and environments for the user [6]. Today’s development of smart homes and indoor environments intended for a person with disabilities is based on the concepts of Ambient Assisted Living (AAL) and Enhanced Living Environments (ELE). The technologies and working principles used in the mentioned concepts are based on modern information and communication technologies such as Cloud Computing (CC) (Fog, Edge, Dew), the Internet of Things (IoT), Machine to Machine (M2M), and diverse types of sensor networks and corresponding sensors [7]. Several different communication devices and systems are currently in daily use, and in most cases, the devices are used for multiple purposes and solve more than just one need [8]. The diversity of technologies available to support the autonomy and independence of people with disabilities offers the possibility of choosing a particular technological solution or set of solutions that are most suitable for their functional abilities [9]. According to the data on the quantity of IoT devices used in smart environments until the year 2025, there are 71 billion installed IoT devices expected, whereas in smartphone environments 12.86 billion devices are expected [10]. Following the development trends in the field of AAL and ELE concepts, the need for the application of diverse types of sensors, robotics, the IoT, and voice assistants to manage them is increasingly emerging [11,12]. The context of the use of AAL and ELE concepts is presented through a literature review, where the authors stated the possibility of use and purpose in closed spaces and the importance of network heterogeneity [13]. The AAL concept, whose aim is to help and provide real-time information to elderly people and persons with disabilities, is also increasingly applied in the design of new services based on decision support systems [14]. The older group of users is becoming increasingly represented in numerous studies, based on the fact that the number of users aged 65+ is increasing. This is also supported by data from the United Nations, where in 2021 there were 761 million elderly people, and it is predicted that this number will reach 1.6 billion in 2050 [15]. The aforementioned fact prompted the authors to design and evaluate the SecurHome TV service, which is based on the interaction of elderly people with their TV receivers as it is one of the most frequently used devices in everyday life [16]. SecurHome TV represents an ecosystem, with a special emphasis on the algorithm for warning users and detecting dangerous situations, contributing to monitoring the activities of an older group of users. For modeling IoT services and delivering real-time information to persons with disabilities, it is possible to use a conceptual model of system architecture applicable to an IoT environment [17]. According to a research paper [18], incorporating IoT technology in a smart home can greatly benefit visually impaired individuals by facilitating their daily activities. This, in turn, enhances the overall autonomy, safety, and comfort of users in their own home environment.

The development of technology such as AI provides the ability to detect and monitor the daily activities of people with disabilities (users) in their homes. In the user’s smart home environment, the following system components are important: appropriate hardware equipment for data collection (sensors and actuators), a communication network (sensor autonomous network), a set of applications and tools for data processing and management, and data storage equipment [5]. The e-care@home system defines certain data sets and the modularity of the smart home system, which enables use regardless of the type of impairment the user has [19]. Also, the application of AI technology and the 6G network is applied in medicine as a closed user space where appropriate IoT services (IoMT) are applied [20]. The application of autonomous and intelligent networks and systems is today also increasingly used indoors, mostly in the field of eHealth and in providing services for the senior persons and persons with disabilities [21,22].

In addition to the application of AI technology and the environment of a smart home for people with disabilities, it is also possible to use it for elderly users who also have certain health problems, such as dementia and an increased risk of falling when moving. For this purpose, the authors propose the improvement of data sets with the aim of a more efficient system for detecting user falls and informing caregivers [23]. AI requires the definition and use of certain types of data sets and thus the creation of user forms for which it is also necessary to define user needs. Daily activities depend on the distinctive characteristics or types of the user’s impairment and an individual approach to solving daily needs is frequently required. For this purpose, to model innovative services in a sustainable ecosystem, it is necessary to clearly define data sets, all to deliver accurate information to the end user and the possibility of predictive information.

User needs are the basis for defining data sets, but also for designing a smart home according to user requirements [8,24,25,26]. There are 624,019 persons with disabilities in the Republic of Croatia, of which 353,550 are male (56.7%), and 270,469 are female (43.3%), thus persons with disabilities make up about 16.0% of the total population. Groups of users and their number in the Republic of Croatia, according to the type of impairment that is the subject of this research, are locomotor system impairment 177,547, speech-voice communication impairment 51,149, visual impairment 20,526, and hearing impairment 17,679 [27].

### 2.2. Defining User Needs in a Smart Home Environment

Research [28] has explored the lifetime home standards (LHTS) of individuals with disabilities. The conclusions are mainly focused on the architectural adaptation of the environment in which the user is located. The research also highlighted the importance of incorporating technological solutions to enhance their living conditions. However, high-tech solutions such as electronic doors and windows remain financially inaccessible to most users. Nonetheless, users expressed that implementing such solutions would improve their quality of life. Safety within the home was also emphasized, particularly the need to detect electric shocks and gas leaks as well as the importance of lighting management. Another piece of research has examined the benefits of home automation systems for visually impaired individuals [29]. The research results point out that there is a strong interest in home automation to increase the autonomy of visually impaired individuals, with lighting control and heating systems being the most prominent areas of focus.

To design the conceptual system architecture, the smart home environment for the visually impaired can be considered through three segments: user needs, the home environment or architecture, as well as the technology used in the home [5]. This approach can also be applied to other groups of people with disabilities, where it is important to show the characteristics of impairment as shown in Table 1.

Based on the type of user impairment, Table 2 defines increased risks depending on the smart home usage scenario (kitchen—K, living room—LR, bedroom—SR, bathroom/toilet—TWC, terrace/balcony—TB, and daily activities—DA).

### 2.3. Defining Data Sets in a Smart Home Environment

Data sets will be defined as elements of the smart home management system based on the above-mentioned increased risks for certain groups of users and usage scenarios. It is also important to emphasize the communications segment that is necessary for the operation of an automated system based on generative AI. The sensors and function tags used in the smart home environment are defined through three subsets, shown in Table 3.

**Figure 2:**
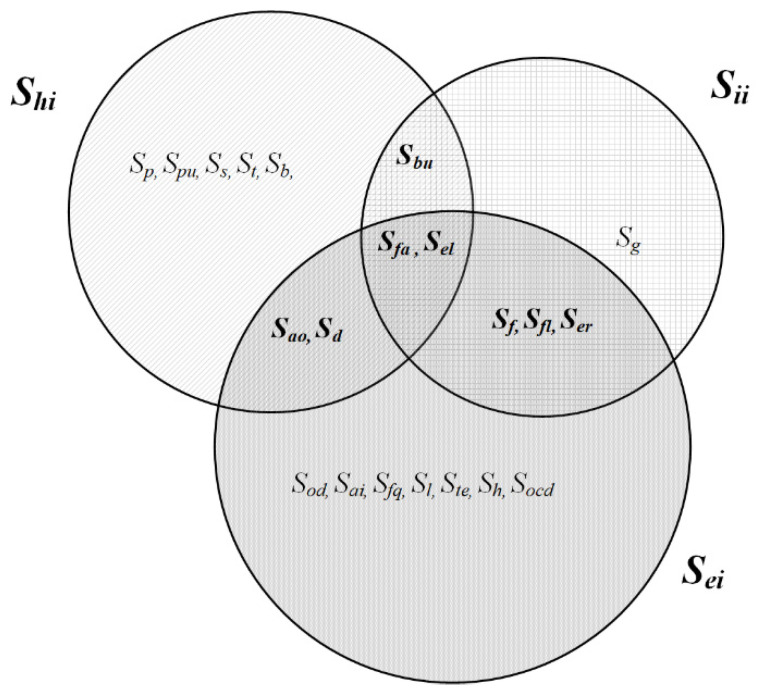
Representation of variables in subsets.

Within each subset, defined sensors collect the data needed to deliver the information requested by the user. Sensors are defined as variables and have their labels, and they are shown with a Venn diagram and a mathematical model (Figure 2) [17]. The conceptual mathematical model for providing information (predictive information) to the user is presented as a union of defined sets, i.e., data collected from around the smart home.

(1) $$S_{shome}=S_{hi}\cup S_{ii}\cup S_{ei}\;\text{or if it is an expansion with additional subsystems}$$

(2) $$S_{shome}=\overset{S}{\underset{i=1}{⋃}}S_{i}$$

from which:

(3) $$S_{hi}=\left\{S_{p},\;S_{pu},S_{s},S_{t},S_{b},S_{bu},\;S_{fa},S_{el},S_{d},S_{ao\;}\right\}$$

(4) $$S_{ii}=\left\{S_{f},\;S_{g},S_{fa},S_{fl},\;S_{el},\;{S_{er},S}_{bu}\right\}$$

(5) $$S_{ei}=\left\{S_{od},\;S_{ai},\;S_{fg},\;S_{l},\;{S_{te\;},\;S_{h\;},S_{d},S_{ao\;},S_{ocd},S_{f},S_{fl},S}_{el},\;S_{fa},\;S_{er}\right\}.$$

Dependence of individual variables in several subsystems is as follows:

(6) $$S_{hi}⋂S_{ii}⋂S_{ei}=\left\{S_{fa},S_{el}\right\}$$

(7) $$S_{hi}⋂S_{ii}=\left\{S_{bu}\right\}$$

(8) $$S_{ii}⋂S_{ei}=\left\{S_{f},S_{fl},\;S_{er}\right\}$$

(9) $$S_{hi}⋂S_{ei}=\left\{S_{ao},S_{d}\right\}$$

The graphical representation in Figure 2 shows the inclusion of individual data (variables) from all these subsystems. Fall detection (*S_fa_*) and electric shock (*S_el_*) are connected to all three subsystems because the delivery of information about the user’s health (state in which they are and type of impairment) and location must be real-time according to the emergency services or the caregivers if this has been previously defined. Burn detection (*S_bu_*) on the user is identified by sensors from the subset of user health (*S_hi_*) and incident information (*S_ii_*) to call emergency services. When a flood (*S_fl_*), fire (*S_f_*), and earthquake (*S_er_*) are detected from the environment where the user is located, a call to the emergency services or the caregiver is also made. To preserve the user’s health (allergic reactions), the sensor of dust (*S_d_*) and air quality in the external environment (*S_ao_*) informs the user about this. All defined variables represent a set of user data necessary for the creation of real-time information and the possibility of predictive user information.

**Figure 3:**
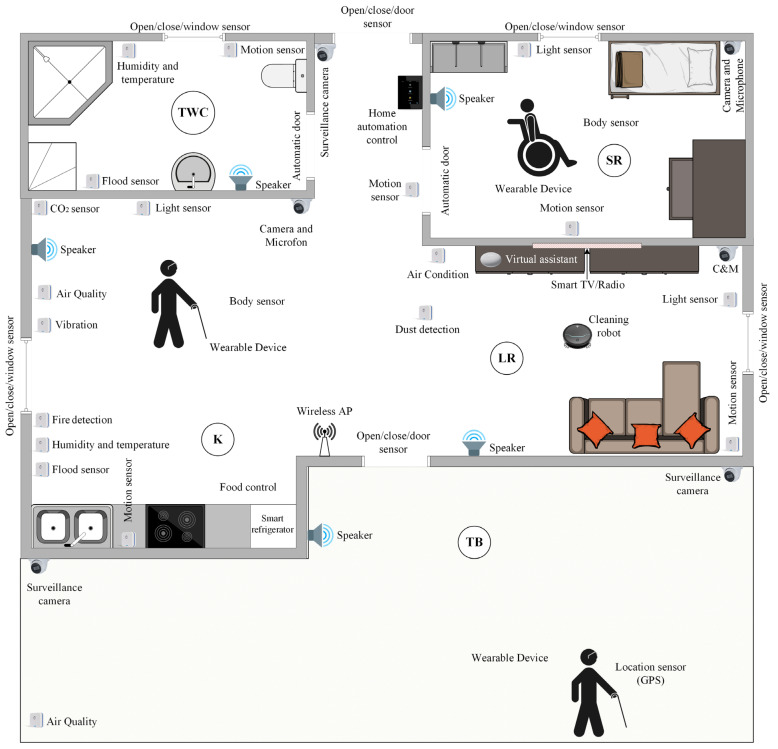
Concept of a smart home for a person with disabilities.

The concept of a smart home for person with disabilities and associated sensors is shown in Figure 3. The figure shows all the sensors listed in Table 3. Inside the smart home, the user can be informed by voice about the time (hours and minutes), reminder management, and environmental detection based on data on incidents such as vibrations (earthquake), flood, etc. The device can be used to control TV and radio devices, refrigerators, vacuum cleaners, air conditioners, front doors, blinds, cameras, and other available IoT devices. It is also possible to provide information to the user about health, during which it is possible to monitor the user’s pulse, pressure, temperature, and the amount of sugar and oxygen in the blood. For this purpose, the user should have a smart bracelet that is equipped with the appropriate sensors. The smart bracelet, with its characteristics, can be modular, i.e., adaptable to the user’s type of impairment. Depending on the type of impairment, appropriate sensors are activated and the format of providing information to the user is adjusted [30]. The connection of the bracelet with the smart home system is also required to create a user base.

Inside the smart home, there are sensors for a safer and more comfortable execution of everyday activities. The virtual assistant provides voice control of smart IoT devices and provides the user with information collected from sensors. Sensor information is delivered upon user request. For the mentioned concept, it is important to use an application solution made according to the guidelines of universal design. The idea of the presented concept is to enable the creation of a user base of the user’s needs to provide accurate and real-time information and predictive user information.

### 2.4. Taxonomy of Communication Technologies and Devices

**Figure 4:**
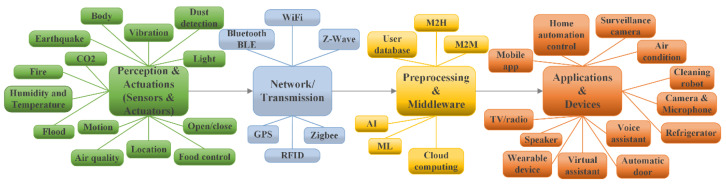
Taxonomy of the used technology and devices.

To apply AI technology, i.e., perception of the environment in which the user is located and for the provision of relevant information, a taxonomy of communication technologies and devices shown in Figure 4 is proposed. The purpose of using AI is to ensure, based on collected data from sensors that monitor everyday situations and movements, that the user’s behaviour and obligations are created by appropriate user patterns as a basis for predictive user information. User forms creation (depending on the type of impairment) defines the basis for modeling IoT services based on AI technology, all to develop a predictive model for information delivery.

For service delivery to the user, the smart home environment is divided into four layers of automation: Perception and Actuations, Network/Transmission, Preprocessing and Middleware, and Applications and Devices. The Perception and Actuations layer represents physical objects that serve to collect information from the user’s environment. The user’s environment is made up of defined scenarios in which they find themself every day (kitchen, living room, bedroom, toilet, and outdoor balcony/terrace), as well as the user’s health and daily needs.

The user’s health condition is monitored via a smart bracelet equipped with body sensors. Body sensors are a group of sensors that collect data on heart rate, pressure, sugar, temperature, and breathing. Also, to monitor the user’s health, a sensor is used to detect burns, electric shock, and sensors that can indicate possible allergic reactions (dust sensor and air quality in the external environment) are used. Sensors that are used to warn about the possible occurrence of an accident and belong to the group for providing incident information have the role of detecting the following: a fire, gas (CO_2_), user fall, a flood, electric shock, an earthquake, and burns. To monitor user activity in all scenarios, sensors are used for objects/obstacles detection, the monitoring of air quality in the indoor environment, food quality, the detection of light intensity, temperature measurements in the home, and measurements of the amount of moisture and dust.

The Network layer or Transmission layer is responsible for the secure transmission of data obtained from the sensor devices to the processing system, i.e., to the Preprocessing and Middleware layer through various network technologies. It consists of an access sublayer that has the role of collecting data from the Perception layer and of sending it to the Internet sublayer. In the access sublayer, which is in the part of the core network, the key role is played by the technologies Z-Wave, Zigbee, Bluetooth BLE, GPS, and RFID. Wi-Fi technology is used to transfer the obtained information from the access sublayer to the next layer, which is Preprocessing and Middleware [5]. GPS technology has the function of determining the user’s position if the user is in the external environment of the smart home or if the user moves away from the home in which they are located (shown in Figure 3). The above is important if the user has some form of dementia or similar impairments.

The Preprocessing and Middleware layer is a software layer that can directly synchronize the services with the appropriate requirements and has a connection to the database. It performs processes such as preparing the information for further processing, data cleaning, standardization, values of data within a dataset, balancing, intelligent routing, and translating network addresses. It is based on CC because of the great benefits it provides, such as scalability and flexibility. In the proposal of this paper, the user data are saved in the user database that is stored in the CC infrastructure. The preparation of information for delivery in the appropriate form is enabled using M2H (Machine–to–Human) and M2M technologies. The ML (machine learning) algorithms enable the intelligent adaptation of applications to changes in the environment, and the AI technology helps to collect data from the devices, predict the user behaviour within the smart home environment, and help to improve data security [5].

Based on the processed data in the Preprocessing and Middleware layer, the Application and Devices layer is responsible for providing end-user services and the proper operation of applications and devices. The identification, processing, and storage of data are performed in CC models depending on the level of service used. In addition to sensors and actuators (*S_n_*_+1_ and *A_n_*_+1_), devices (*D_n_*_+1_) are also used, which by their characteristics belong to the group of IoT devices and have been integrated into the smart home system. Devices include smart robots, air conditioners, automatic doors at the home and room entrances, a smart TV, a refrigerator, cameras, a virtual assistant, and smart home management systems. Application solutions on smartphones and smart home management systems are also used. An API (Application Programming Interface) is used to provide the appropriate form of information that is defined through the user profile (degree of impairment, etc.).

## 3. Conceptual System Architecture

**Figure 5:**
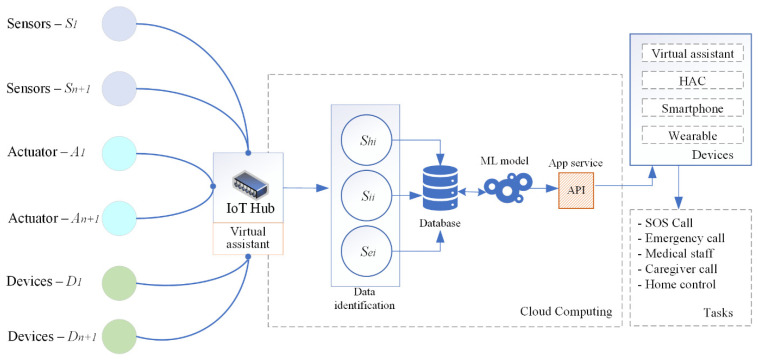
Conceptual architecture of the data collection and processing system.

According to the defined sensors and the communication network used in the smart home environment, and the defined scenarios of the application of the smart home service, Figure 5 shows the conceptual architecture of the data collection and processing system. The communication of the elements is based on the AAL/ELE concept, the main purpose of which is to work in closed spaces adapted to user needs [12,13,31].

**Figure 6:**
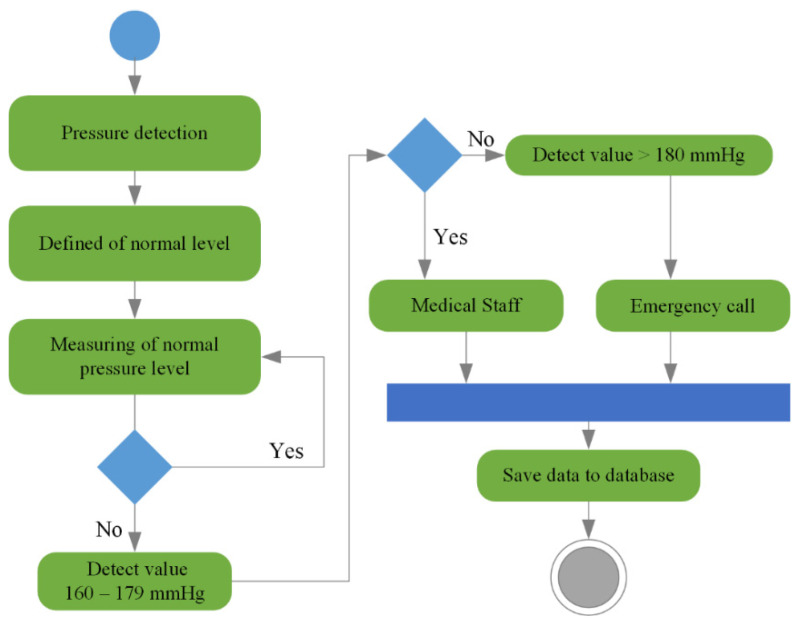
The process of detecting an increased level of pressure in the user.

The role of sensors and actuators (*S_n_*_+1_ and *A_n_*_+1_) is to collect data from the user’s environment depending on the scenario in which the smart home service is used. Devices (*D_n_*_+1_) used in the smart home environment also generate data sets in the IoT hub and forward them to the CC, whose role is to collect, identify, and process data. The interaction between all levels of communication towards the CC depends on the service-level agreement (SLA), which must satisfy the corresponding level of service. The data are stored in the database depending on the identification of the associated set (function dependencies—*S_hi_*, *S_ii_*, and *S_ei_*). An example of detecting an elevated pressure level in a user and performing tasks is shown in Figure 6.

When detecting pressure, it is important to set the pressure levels (normal–high). The mentioned levels also represent a weighting factor that is crucial for the execution of tasks and activities. One of the tasks called emergency call consists of several activities (activities such as contacting emergency services in case of fire, flood, fall, injury) that can be performed in different usage scenarios (K, LR, SR, TWC, TB, DA) depending on the type of user impairment. At the same time, the activities represent the way and the possibility of delivering information to the user or one of the defined stakeholders, and their sets are defined as tasks that are performed through specific devices.

Through the Application Programming Interface (API), information is displayed to the user/stakeholder in a specific format. Each piece of information is adapted to the user (depending on the type of impairment) using M2H communication in an acceptable form. To collect data on users, it is possible to use a separate database where the user data needed to define user profiles (type of impairment and method of data delivery) are located. The content is adjusted based on the user’s characteristics and the functionalities selected by the individual user and is presented in Table 4.

For persons with locomotor impairments, it is important to consider all the above-mentioned guidelines if it is an additional type of impairment; however, for persons who move with the help of wheelchairs, the aspect of providing accurate information and communication with the caregiver or emergency services is important. When identifying an incident situation, a person must have the simplest viable way out of the home environment. People who do not have speech or hearing problems but have a visual impairment or are physically impaired and use a wheelchair can use the services of a voice assistant in a smart home environment. Its role is the ability to manage IoT devices in the home and predictive user information.

## 4. Defining User Profiles

Based on the defined data sets and depending on the type of user impairment, user profiles are defined as shown in Table 5. The information is crucial for the design and development of personalized user services with the aim of providing accurate and real-time information in the user’s home environment.

The user profiles shown in Table 5 are divided into four categories: user characteristics, user needs, methods of collecting data and information, and the possibility of personalized messages. In the group of user characteristics, it is important to know what type of impairment is involved and what level it is (e.g., 75% vision impairment or less with no colour recognition). In this group, it is also important if, for example, it is a case of a deaf–blind person for whom it is necessary to additionally provide communication modules. Also important is the user’s level of IT literacy, how much they use today’s smartphones, etc. In this section, the required level of assistance support is also important, whether it is fully automated or partial, e.g., for full automation in a smart form, voice, visual, and tactile information are required, while for partial automation, the user could perform certain activities. Additional specific information relates, for example, if the user has a problem holding the device or speaks more slowly, so that adaptation is also required in that area.

Information about user needs is focused on monitoring daily activities (morning, day, night) in a 24 h time interval or at a specific time, the possibility of using medical staff or a caregiver requested by the user themself and communicating with them, and monitoring the increased risk of other forms of injury (falls or something similar). If the user requires the ability to use diverse types of communicators, for example sign language, this is also essential information in creating the profile. The user’s level of mobility, navigation, and accessible environment are information that increase the user’s autonomy in their home.

Data and information can be collected from the sensors shown in this paper and depending on the example in the smart home at the Figure 3. In addition, it is possible to collect data on user behaviour and use data from health records if available.

For creating predictive information, the way of personalized messages is important. Personalized reminders aim to provide information based on the user’s daily routines, e.g., taking medication, rest, exercise, etc. Working with automated devices implies the possibility of voice or some other form to be used with devices in the home to provide information about planned events, e.g., if a trip to the doctor is planned, etc., with the user receiving a notification about it.

When delivering information in real time, it is necessary to consider the mechanisms for providing notifications, and to enable:

- The possibility of synchronizing the information delivery system, which must be consistent with the environment in which the user is located;
- The possibility of providing information (notifications) in different modalities depending on the type of user impairment (visual, audio, or haptic);
- That the user information service is designed according to the principles of universal design, which allows avoiding straining the user with excessive or distracting notifications.

The importance of using individual mechanisms in the system of providing information to users is also stated by the authors in the paper [32]. In the paper, an analysis of user perceptions regarding the provision of information used by users in different scenarios was made, during which various measurement variables were used (engagement, distraction, awareness, and emotional effort). With the approach, the authors tried to create a balanced user experience in the observed scenario (delivery of synchronized information related to TV content, and informing users via visual, audio, and haptic stimuli).

## 5. Testing and Performance Results of Sensors in a Smart Home Environment

**Figure 7:**
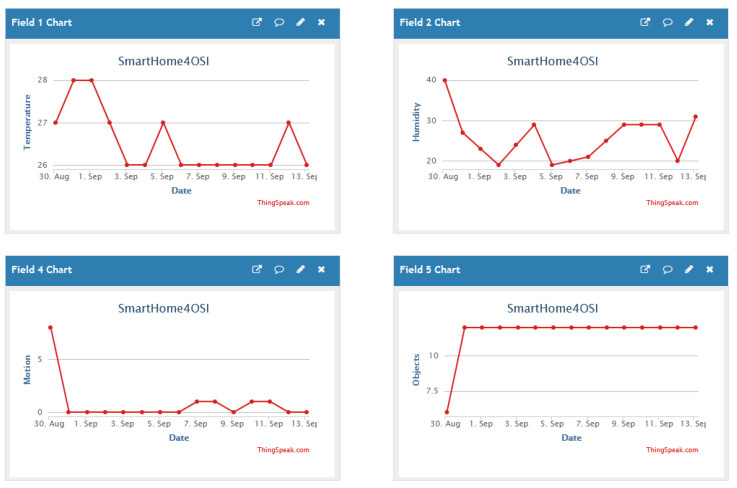
Thingspeak environment for monitoring collected data from sensors.

To verify the effectiveness of the proposed mathematical model, which aims to define information for users that are in smart home environment, specific sensors and IoT equipment were utilized. The data were collected and processed in the Thingspeak cloud environment for data storage and continuous monitoring of information from the user’s environment. Figure 7 shows the Thingspeak environment and the data collection time in laboratory conditions. Data were collected at average intervals of 15 [s]. Each record includes a unique identifier (id), time interval between measurements, sensor value, latitude, longitude, elevation, and status (2023-08-29T16:14:05+02:00, 15, 28.00000, 82.39999, 40.00000).

The collected values are shown in Figure 7, for example, the temperature value from 26 °C to 28 °C, the humidity amount from 20% to 40%, fall detection (motion), and recognition of objects at a certain distance from 3 [m] to 15 [m]. The equipment used for simulation and testing is shown in Table 6. The test was conducted in the Laboratory of Development and Research of Information and Communication Assistive Technology at the Faculty of Transport and Traffic Sciences, University of Zagreb.

**Figure 8:**
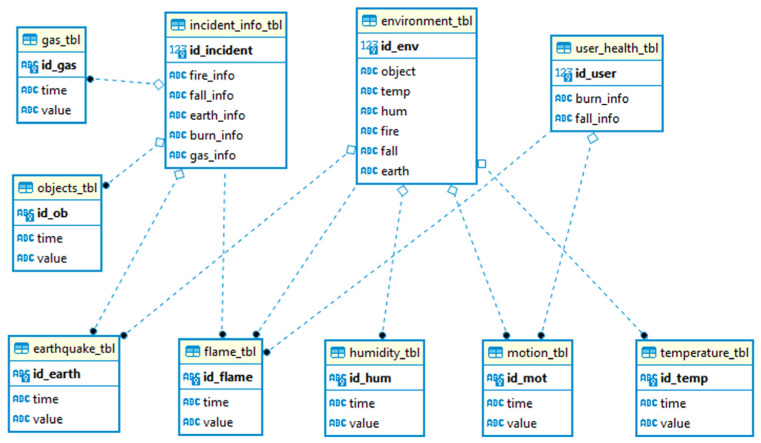
Relations within the database.

The sensors collected various values including fire, burn, CO_2_, earthquake, object, temperature, humidity, and fall. These values were then stored in the Thingspeak cloud environment and later in the MYSQL database, as shown in Figure 8. The database was created based on the mathematical model that described the type of data and values. The tables that collected the sensor data were linked to the table responsible for storing and delivering information to the end user.

To deliver information in a suitable format for a specific user, such as a blind or partially sighted person with unique characteristics, an appropriate API must be defined in the middleware layer. In this case, an API can be used to receive data and send configuration values to an API that delivers information in the appropriate format for the user’s device, regardless of the device type.

Testing of sensor performance and storage of collected data according to defined sets *S_hi_*, *S_ii_*, and *S_ei_* showed the importance of storing individual values in precisely defined sets. These sets are essential for defining user profiles, which are necessary for the development of ML models. For instance, in a 15 day period, 80,398 data points were collected. Using a conceptual mathematical model and defined variables, the value of generating information about the fire is shown:

*S_info_fire_ = *{*S_ii_*, *S_ei_*, *S_hi_*}. (10)

Based on the information above, when reporting a fire, incident information (*S_ii_*) is provided along with the ability to call emergency services. The user’s environment (*S_ei_*) also provides information about the user’s location within different scenarios (K, LR, SR, TWC, TB, DA) and whether the user is in danger (*S_hi_*). This method has been found to be highly successful in storing data based on distinct variables and sets, enabling efficient delivery to the user and possible interest groups (stakeholders).

Based on the defined mathematical model for providing predictive information, data collection and storage testing according to the proposed model was conducted. In addition, an in-depth analysis of the papers presented through the literature review was also conducted (Table 7). At the end of the table, the contribution of the conducted research and testing to this paper is presented.

The analyzed papers show the following categories: data collection methods, user data identification, the need for API development, and the existence of a database structure. The analyzed categories are important from the aspect of creating user profiles, all with the aim of creating a sustainable ecosystem in which the service of predictive informing of smart home users is developed. It is evident from the analyzed papers that some papers do not have clearly defined data necessary for the development of the service, or at least data sets, and only some works emphasize the importance of using APIs, which have a clear role in providing relevant information. It is also evident that only one paper has a presentation of the database structure. Also, as shown in Table 7, the article [19] has similarity to this research in all categories. However, the contribution of this paper is manifested through the following:

- The literature review enabled the identification of insights and methodological approaches that are the basis for understanding the issues of data collection and processing. The above is crucial for defining the user profiles and data sets presented in the paper;
- The data sets use relevant data from the user’s environment, and the data are collected from a larger sensor database, which is shown by a mathematical model;
- The mathematical model allows the display of all data regardless of the type of user (a person with disabilities, an older user group, etc.), which enables the modularity of the service’s operation to provide predictive information to the end user.

The above contributions can provide flexibility and long-term scalability to the system and show how the proposed architecture allows for rapid adaptation to different scenarios and end-user needs. Data collection in this paper was performed with the aim of ensuring the accuracy and reliability of the system, and the focus is on validation in laboratory conditions, which is shown by testing the operation of the sensors and the database. This approach allows for the identification and resolution of potential technical issues before implementation in real-world conditions and creates a solid foundation for later experiments in real-world conditions. The use of APIs in this research is the basis for providing predictive information depending on the type of user. The database must contain key data about user needs (shown in Table 1) in which the provision of real-time information is delivered via the API interface.

## 6. Conclusions

This paper proposes a method of data collection as a basis for creating a user knowledge base for the purpose of predictive user information. From the presented data collection testing, it is possible to store data in precisely defined databases, which is the basis for designing new innovative services. With an innovative approach, it is possible to create individual or personalized information depending on the user profiles presented in this paper.

The development of assistive technologies and the application of AI to help people with disabilities today can be said to be not just a type of device, but a set of methods, services, and an entire ecosystem that must be sustainable and accessible. Services based on IoT technology and AI can raise the user’s level of quality of life, which increases the degree of mobility and involvement in all spheres of social life and everyday life. Designing and modeling such systems should be viewed according to user needs and based on that, to create a sustainable and accessible ecosystem. The possibilities provided today by information and communication networks, and technologies such as AI, ML, and autonomous systems, can create a faster and more efficient system for the user. Such possibilities provide predictive user information and interaction with the environment in which they are located.

Based on the proposed taxonomy from this paper, it is possible to create a smart home for a person with disabilities depending on the type and degree of impairment, which aims to achieve a more pleasant and safer everyday life. Based on the displayed interaction between all elements, it is possible to provide the user with all information from the home environment, information about the user’s health (pulse, temperature, pressure, sugar, etc.), and the possibility of calling the emergency services or the caregiver in case of incident. Management of sensors, actuators, and devices in the home is possible through speech using a virtual assistant, which has the potential to improve the mood of the user in his home, if it is a person with impaired vision or a person with physical disabilities. People with voice–speech difficulties and people with a hearing impairment must receive adapted video–visual information to also provide them with a higher quality of life and independence.

The solution described in this paper is an assistive technology model that includes a device, user, service, and environment, providing a personalized smart home experience. With relevant devices and sensors gathering specific data sets, defined by a mathematical model, this solution serves as a vital sub-log for potential application of AI technologies. Laboratory testing has confirmed the significance of defining data sets in generating user-friendly information to the end user. In future development, an API interface should be defined for the sensor to segment and merge data based on user requirements. Future research will be focused on the development of ML models, verification of model effectiveness (simulation and validation), and the creation of a user knowledge base, all to model new IoT services for predictive user information. Future research should also contribute to creating a higher level of quality of life, and thus an improved mood, which can be proven by appropriate methods of measuring user satisfaction. By using different multi-criteria-decision-making approaches, it is possible to select the appropriate IoT components in a smart home and the personalized services themselves.

## Figures and Tables

**Table 1:** User characteristics according to the type of impairment.

Type of Impairment	Characteristics
Blind and partially sighted	slow and uncertain movement difficulties with planning, organizing time, materials, and tasks “fear” of light inability to distinguish colours or shades of colours and letter sizes spatial disorientation limited living space
Locomotive system	physical and architectural barriers free space for wheelchairs to turn around in the room limited living space
Speech-voice communication	speech impairments voice impairment the problem of reading, writing, and arithmetic
Hearing	limited and poor communication problem using speech limited living space avoiding social activities concentration difficulties weaker ability to maintain balance weaker motor speed and coordination

**Table 2:** Display of increased risks depending on the type of impairment and the scenario of using the smart home service.

Type of Impairment	Usage Scenario	Increased Risks
Blind and partially sighted	K	fire during food preparation uncontrolled gas leakage injuries and burns flood electric shock
	LR	obstacles and possible injuries inability to locate individual elements fall
	SR	obstacles and possible injuries inability to locate individual elements
	TWC	flood electric shock injuries and falls
	TB	injuries and falls
	DA	health status monitoring incorrect information using the information service
Locomotive system	K	uncontrolled gas leakage injuries and burns flood electric shock unavailability of elements and injuries
	LR	obstacles and possible injuries fall poor availability of elements
	SR	obstacles and possible injuries fall
	TWC	injuries and falls electric shock flood
	TB	injuries and falls
	DA	health status monitoring incorrect information using the information service
Speech-voice communication	All scenarios	panic and poor reaction to possible incident situations
Hearing	All scenarios	concentration

**Table 3:** The range of functional dependencies required to deliver information at the user’s request.

Range of Function Dependence	User Health (*S_hi_*)	Incident Information (*S_ii_*)	User Environment (*S_ei_*)
Sensors and function tags	Pressure (*S_p_*) Pulse (*S_pu_*) Sugar (*S_s_*) User’s temperature (*S_t_*) Breathing (*S_b_*) Burn (*S_bu_*) Fall (*S_fa_*) Electric shock (*S_el_*) Air quality (external) (*S_ao_*) Dust (*S_d_*)	Fire (*S_f_*) Gas (*S_g_*) Fall (*S_fa_*) Flood (*S_fl_*) Electric shock (*S_el_*) Earthquake (*S_er_*) Burn (*S_bu_*)	Object detection (*S_od_*) Air quality (indoor) (*S_ai_*) Food quality (refrigerator) (*S_fq_*) Light intensity (*S_l_*) Temperature (*S_te_*) Humidity (*S_h_*) Dust (*S_d_*) Air quality (outside) (*S_ao_*) Open/close door (*S_ocd_*) Fire (*S_f_*) Flood (*S_fl_*) Electric shock (*S_el_*) Fall (*S_fa_*) Earthquake (*S_er_*)

**Table 4:** Form of providing information depending on the type of disability.

Type of Impairment	Form of Providing Information
Visually impaired person	Easy to use Designed according to universal design guidelines Using accessibility options for text and background customization Adaptable for TXT option Enabled access to use voice control Recognition of objects through the camera SOS option High accuracy in voice recognition in text
Voice–speech communication difficulties	Adaptable content for visual information Through video visual content, Possibility of using sign language Adjusting the intensity of the speaking speed Easy to use Designed according to universal design guidelines Easy to provide technical assistance SOS option
Hearing-impaired person	Adaptable content for visual information Through video visual content Possibility of using sign language Easy to use Designed according to universal design guidelines Adjusted for the possibility of using light effects SOS option

**Table 5:** User profile elements.

User Characteristics	User Needs	Data Collection Methods	Type of Personalized Messages
Type of impairment Level of support (partial or full assistance) Level of knowledge about using technology Health information Additional specific information depending on the type of impairment	Daily activities Use of medical personnel Prone to falls or other forms of risk Need for different forms of communicators Mobility and navigation Accessibility	Activity tracking sensors Behaviour tracking Health monitoring capabilities	Personalized reminders Automated devices and elements in the home environment Anticipation of potential problems (falls, etc.) Notifications about planned events Warning messages about potential dangers Voice and visual messages Communication with a caregiver or another person

**Table 6:** Display of equipment used for the purpose of simulating data collection in a smart home.

Equipment	The Role
Sensor LM393—Adafruit Industries, New York, NY, USA	Detection of fire in the apartment or burns
Sensor MQ-2—Arduino, Monza, Italy	CO_2_ gas level detection
Sensor SW-420—Arduino, Monza, Italy	Earthquake detection
Sensor HC-SR04—Arduino, Monza, Italy	Object detection
Sensor DHT11—Arduino, Monza, Italy	Measurement of temperature and humidity levels
Sensor PI-HCSR501—Arduino, Monza, Italy	Fall detection
Arduino Mega 2560Rev3—Arduino, Monza, Italy	Microcontroller for connecting sensors and computers
Arduino Ethernet Shield—Arduino, Monza, Italy	A component for connecting and sending data to the Cloud
Thingspeak—The MathWorks, Inc., Natick, MA, USA	Data collection, storage, and real-time monitoring

**Table 7:** In-depth analysis of research methods.

Papers	Research Topic	Data Collection Method	User Data Identification	Importance of API Development	Database Structure Overview
[3]	Overview and categorization of existing devices (AT)	Analysis of previous research results	Yes (partially)	No	No
[6]	Analysis of device operation and smart home functionality	Analysis of previous research results	Yes (partially)	No	No
[8]	Analysis of communication technologies for the smart home	Analysis of previous research results	Yes (no details)	No	No
[9]	Analysis of the application of assistive technologies in a smart home	Analysis of previous research results	Yes (no details)	Yes	No
[11]	Development of a service for the rehabilitation of users	Collecting data from sensors	Yes	Yes	No
[12]	Overview of architectures for assisted living	Analysis of previous research results	Yes (no details)	Yes	No
[13]	Development of AAL environment and presentation of technologies	Collecting data from sensors	Yes	Yes	No
[16]	Development of a notification service in a smart home	Data collection via forms	Yes	Yes	No
[18]	Exploring the challenges and opportunities of a smart home for the visually impaired	Analysis of previous research results	Yes (no details)	Yes	No
[19]	Development of an e-care system for the elderly	Collecting data from sensors	Yes	Yes	Yes
[20]	Development of a framework for the application of fog computing in eHealth	IoT device data collection	No	No	No
[23]	Activity classification for elderly person using ML	Data collection from radar sensors	No	No	No
[24]	Analysis of user needs in nursing homes	Analysis of previous research results	No	No	No
[25]	Online infrastructure design for collecting data on people with disabilities	Conducting surveys and interviews	Yes	No	No
[28]	Analysis of the lifestyle of visually impaired people at home	Conducting interviews	Yes (no details)	No	No
[29]	Research of technologies in the function of home automation for visually impaired people	Online survey and interviews	Yes	No	No
Our Research	Defining user profiles for the purpose of predictive information in a smart home	Analysis of the results of previous research and collection from sensors	Yes	Yes	Yes

## Data Availability

Data are contained within the paper.

## References

[1] World Health Organization Blindness and Vision Impairment. https://www.who.int/news-room/fact-sheets/detail/blindness-and-visual-impairment.

[2] Statista Market Insights (2024). Number of Users of Smart Homes Worldwide from 2019 to 2028.

[3] Mashiata M., Ali T., Das P., Tasneem Z., Badal M.F.R., Sarker S.K., Hasan M.M., Abhi S.H., Islam M.R., Ali M.F. (2022). Towards Assisting Visually Impaired Individuals: A Review on Current Status and Future Prospects. Biosens. Bioelectron. X.

[4] Periša M., Peraković D., Zorić P., Janošević D. (2019). Challenges of Assistive Technologies Implementation into Industry 4.0: A Review. Proceedings of the The Seventh International Conference Transport and Logistics.

[5] Periša M., Cvitić I., Zorić P., Grgurević I., Knapčíková L., Peraković D. (2023). Concept, Architecture, and Performance Testing of a Smart Home Environment for the Visually Impaired Persons. Proceedings of the EAI MMS 2022—7th EAI International Conference on Management of Manufacturing Systems.

[6] Maswadi K., Ghani N.B.A., Hamid S.B. (2020). Systematic Literature Review of Smart Home Monitoring Technologies Based on IoT for the Elderly. IEEE Access.

[7] Dobre C., Mavromoustakis C.X., Garcia N.M., Mastorakis G., Goleva R.I., Dobre C., Mavromoustakis C.X., Garcia N., Goleva R.I., Mastorakis G. (2017). Introduction to the AAL and ELE Systems. Ambient Assisted Living and Enhanced Living Environments: Principles, Technologies and Control.

[8] Jamwal R., Jarman H.K., Roseingrave E., Douglas J., Winkler D. (2022). Smart Home and Communication Technology for People with Disability: A Scoping Review. Disabil. Rehabil. Assist. Technol..

[9] Colnar S., Dimovski V., Grah B., Rogelj V., Bogataj D. Smart Home Supporting Integrated Health and Care Services for Older Adults in the Community: Literature Review and Research Agenda. Proceedings of the 2020 24th International Conference on System Theory, Control and Computing (ICSTCC).

[10] Cvitić I., Peraković D., Periša M., Gupta B. (2021). Ensemble Machine Learning Approach for Classification of IoT Devices in Smart Home. Int. J. Mach. Learn. Cybern..

[11] Bisio I., Garibotto C., Lavagetto F., Sciarrone A. (2019). When EHealth Meets IoT: A Smart Wireless System for Post-Stroke Home Rehabilitation. IEEE Wirel. Commun..

[12] El Murabet A., Abtoy A., Touhafi A., Tahiri A. (2020). Ambient Assisted Living System’s Models and Architectures: A Survey of the State of the Art. J. King Saud. Univ. Comput. Inf. Sci..

[13] Cicirelli G., Marani R., Petitti A., Milella A., D’Orazio T. (2021). Ambient Assisted Living: A Review of Technologies, Methodologies and Future Perspectives for Healthy Aging of Population. Sensors.

[14] Kunnappilly A. (2021). Modeling and Formal Analysis of E-Health Systems. Ph.D. Thesis.

[15] United Nations (2023). World Social Report: Leaving No One behind in an Ageing World.

[16] Abreu J., Oliveira R., Garcia-Crespo A., Rodriguez-Goncalves R. (2021). TV Interaction as a Non-Invasive Sensor for Monitoring Elderly Well-Being at Home. Sensors.

[17] Periša M., Marković G., Kolarovszki P., Madleňák R. (2019). Proposal of a Conceptual Architecture System for Informing the User in the IoT Environment. Promet. Traffic Transp..

[18] De Oliveira G.A.A., Oliveira O.D.F., de Abreu S., de Bettio R.W., Freire A.P. (2022). Opportunities and Accessibility Challenges for Open-Source General-Purpose Home Automation Mobile Applications for Visually Disabled Users. Multimed. Tools Appl..

[19] Köckemann U., Alirezaie M., Renoux J., Tsiftes N., Ahmed M.U., Morberg D., Lindén M., Loutfi A. (2020). Open-Source Data Collection and Data Sets for Activity Recognition in Smart Homes. Sensors.

[20] Sodhro A.H., Zahid N. (2021). AI-Enabled Framework for Fog Computing Driven E-Healthcare Applications. Sensors.

[21] Donnici R., Coronato A., Naeem M. A Self-Learning Autonomous and Intelligent System for the Reduction of Medication Errors in Home Treatments. Proceedings of the Intelligent Environments 2021: Workshop Proceedings of the 17th International Conference on Intelligent Environments.

[22] Aski V.J., Dhaka V.S., Kumar S., Verma S., Rawat D.B. (2022). Advances on Networked EHealth Information Access and Sharing: Status, Challenges and Prospects. Comput. Netw..

[23] Taylor W., Dashtipour K., Shah S.A., Hussain A., Abbasi Q.H., Imran M.A. (2021). Radar Sensing for Activity Classification in Elderly People Exploiting Micro-Doppler Signatures Using Machine Learning. Sensors.

[24] Zimmermann G., Ableitner T., Strobbe C. User Needs and Wishes in Smart Homes: What Can Artificial Intelligence Contribute?. Proceedings of the 2017 14th International Symposium on Pervasive Systems, Algorithms and Networks & 2017 11th International Conference on Frontier of Computer Science and Technology & 2017 Third International Symposium of Creative Computing (ISPAN-FCST-ISCC).

[25] Park J.S., Bragg D., Kamar E., Morris M.R. (2021). Designing an Online Infrastructure for Collecting AI Data from People With Disabilities. Proceedings of the 2021 ACM Conference on Fairness, Accountability, and Transparency.

[26] Samim A. (2023). A New Paradigm of Artificial Intelligence to Disabilities. Int. J. Sci. Res..

[27] Benjak T. (2021). Izvješće o Osobama s Invaliditetom u Republici Hrvatskoj.

[28] Rooney C., Hadjri K., Mcallister K., Rooney M., Faith V., Craig C. (2018). Experiencing Visual Impairment in a Lifetime Home: An Interpretative Phenomenological Inquiry. J. Hous. Built Environ..

[29] Leporini B., Buzzi M. (2018). Home Automation for an Independent Living. Proceedings of the Proceedings of the 15th International Web for All Conference.

[30] Peraković D., Periša M., Sente R.E., Bijelica N., Zorić P., Brletić L., Bucak B., Ignjatić A., Mišić V., Papac A., Balog M., Knapcikova L., Soviar J., Dorcak P., Pollak F., Caganova D., Fazio P., Aydin K. (2016). Information and Communication System for Informing Users in Traffic Environment—SAforA. Proceedings of the 2thEAI International Summit, Smart City 360°.

[31] Goleva R.I., Garcia N.M., Mavromoustakis C.X., Dobre C., Mastorakis G., Stainov R., Chorbev I., Trajkovik V. (2017). AAL and ELE Platform Architecture. Ambient Assisted Living and Enhanced Living Environments.

[32] Almeida P., Abreu J., Silva T., Duro L., Aresta M., Oliveira R. Notification Mechanisms In Second-Screen Scenarios—Towards a Balanced User Experience. Proceedings of the 7th International Conference on Intelligent Technologies for Interactive Entertainment.

